# The relationship between intake of fruits, vegetables and dairy products with overweight and obesity in a large sample in Iran: Findings of STEPS 2016

**DOI:** 10.3389/fnut.2022.1082976

**Published:** 2023-01-17

**Authors:** Mehran Nouri, Zainab Shateri, Shiva Faghih

**Affiliations:** ^1^Department of Community Nutrition, School of Nutrition and Food Sciences, Shiraz University of Medical Sciences, Shiraz, Iran; ^2^Student Research Committee, Shiraz University of Medical Sciences, Shiraz, Iran; ^3^Health Policy Research Center, Institute of Health, Shiraz University of Medical Sciences, Shiraz, Iran; ^4^Student Research Committee, Ahvaz Jundishapur University of Medical Sciences, Ahvaz, Iran; ^5^Nutrition Research Center, Shiraz University of Medical Sciences, Shiraz, Iran

**Keywords:** fruits, vegetable, dairy, STEPS, overweight, obesity

## Abstract

**Background:**

The present study aimed to investigate the association between fruits and vegetables (FVs) and dairy product intake with body weight based on the data from the nationwide Stepwise approach to surveillance (STEPS) survey in Iran.

**Methods:**

STEPS is a national-based cross-sectional study conducted on 3,0541 people selected by stratified cluster random sampling in Iran from April to November 2016. The outcome of the current study was body mass index (BMI) which was classified as normal weight, underweight, overweight, and obese. Also, as exposure variables, dietary intakes of fruits, vegetables and dairy products were extracted from the STEPS questionnaires. Multinomial logistic regression was used to evaluate the association between dairy products, FVs consumption, and BMI category in the crude and adjusted models.

**Results:**

In the adjusted model, we observed 41% [odd ratio (OR) = 0.59; 95% confidence intervals (CI): 0.50, 0.68, P < 0.001], 27% (OR = 0.73; 95% CI: 0.62, 0.84, *P* < 0.001), and 26% (OR = 0.74; 95% CI: 0.63, 0.87, *P* < 0.001) lower odds of being overweight, and 46% (OR = 0.54; 95% CI: 0.46, 0.64, *P* < 0.001), 29% (OR = 0.71; 95% CI: 0.60, 0.84, *P* < 0.001), and 21% (OR = 0.79; 95% CI: 0.65, 0.95, *P* = 0.014) decrease in obesity odds among the participants who consumed 1, 2, and more than 2 servings of fruits per day in comparison to less than one serving, respectively. Also, we observed participants who consumed 1, 2, and more than 2 servings in comparison to less than one serving of dairy products per day had 31% (OR = 0.69; 95% CI: 0.58, 0.81, *P* < 0.001), 23% (OR = 0.77; 95 %CI: 0.65, 0.91, *P* = 0.002), and 21% (OR = 0.79; 95% CI: 0.67, 0.94, *P* = 0.011) lower odds of being overweight and 47% (OR = 0.53; 95% CI: 0.44, 0.64, *P* < 0.001), 36% (OR = 0.64; 95% CI: 0.53, 0.77, *P* < 0.001), and 32% (OR = 0.68; 95% CI: 0.56, 0.83, *P* < 0.001) lower odds of obesity, respectively. In addition, compared to participants who consumed less than 2 servings of vegetables per day, participants who consumed 2, 3, and more than 3 servings had 40% (OR = 0.60; 95% CI: 0.47, 0.76, *P* < 0.001), 29% (OR = 0.71; 95% CI: 0.56, 0.90, *P* = 0.006), and 26% (OR = 0.74; 95% CI: 0.57, 0.96, *P* = 0.027) lower odds of being overweight, respectively. Furthermore, we observed 36% lower odds of obesity among participants who ate 2 servings of vegetables per day compared to less than 2 serving (OR = 0.64; 95% CI: 0.49, 0.84, *P* = 0.002).

**Conclusion:**

Our findings showed that intake of FVs and dairy products is associated with a healthier weight status in adults. Further studies are needed to confirm these findings.

## Introduction

Obesity is one of the most serious health problems in Asian countries, including Iran (1). Iran is a developing country, experiencing urbanization, cultural changes, and economic and social transition, all of which can affect the prevalence of overweight and obesity (2). The latest World Health Organization (WHO) report in 2010 showed that more than 50% of Iranian adults are overweight or obese (3).

Obesity is an epidemic causing serious health consequences (4), including diabetes, dyslipidemia, and hypertension (5). Diet has received the most attention among the lifestyle behaviors affecting obesity (6). Fruits and vegetables (FVs) are high in water and fiber and low in energy density, so their consumption is suggested to prevent obesity (7). Various mechanisms support the beneficial effects of FVs consumption on body weight. FVs have a satiety effect, which reduces the calorie intake (8, 9). They are also an excellent alternative for high-calorie foods (10, 11). FVs could adjust the glycemic load of the diet due to their fiber content, which affects hormonal changes during fed state (12, 13). Studies have shown different results regarding the relationship between FVs intake and body weight (10, 14–16). Some studies have indicated a negative relationship between FVs intake and body weight (17, 18), while others have reported no association (19, 20).

Recently, there has been much scientific interest and debate on the potential link between dairy consumption and body weight regulation (21). A cross-sectional study found that higher consumption of dairy products was associated with a lower odds of obesity (22). Also, a review study by Dougkas et al. (23) showed that dairy products are inversely related or not related to obesity and adiposity indicators in children. Further, a meta-analysis of clinical trials indicated that increasing dairy intake did not affect fat loss and body weight in studies without energy restriction or long-term studies. Still, dairy product consumption may exert a modest effect on weight loss in the randomized clinical trial with energy restriction or short-term studies (24). Beside FVs intake, there is much scientific interest and debate on the potential link between dairy consumption and body weight regulation (21). Dairy products reduce the risk of obesity due to their components including conjugated linoleic acid, branched-chain amino acids, medium-chain fatty acids, protein, vitamin D, and calcium (25). It has been shown that calcium ion inside the cell affects adipocyte metabolism (26, 27). So that, increasing calcium intake reduces ionic calcium concentration in adipocytes by suppressing the parathyroid hormone. Decreasing the concentration of ionized calcium increases the oxidation of fats, inhibits *de novo* lipogenesis, and stimulates lipolysis (23). There are various studies on the relationship between dairy consumption and obesity. Some studies reported an inverse association between dairy consumption and body weight (28–30) and some studies indicated a positive association (31, 32).

Considering the high prevalence of overweight and obesity in Iran, dietary strategies are necessary to maintain a healthy weight. This study investigated the effect of specific food groups on body weight in a large sample, Finally, given the contradictory findings regarding the relationship between the intake of FVs and dairy with body weight, the present study aimed to investigate the association between FVs and dairy intake with obesity using the data from a nationwide Stepwise approach to surveillance (STEPS) survey in Iran. The findings of this study can identify effective food groups for maintaining a healthy weight.

## Materials and methods

### Design and sampling

This national-based cross-sectional study was done by the Non-Communicable Diseases (NCD) Research Center of Tehran University of Medical Sciences between April and November 2016. It covered all provinces of Iran except Qom (33). According to WHO advice for changing the questionnaire by regional interest, the adapted type of WHO-based STEPS (STEPwise method to surveillance) questionnaire was used for data collection (34). The questionnaire’s validity and reliability have been assessed before (33).

Medical history, dietary intake and general questions were asked in the questionnaire. Information divided to personal, lifestyle, home situation, socioeconomic status, occupation, anthropometric indices, smoking status, physical activity, NCDs history, and etc. Out of 31,050 participants selected by stratified cluster random sampling, finally, 30,541 eligible individuals completed the STEPS questionnaire. In the first phase, socio-demographic characteristics, medical history, and lifestyle data (e.g., physical activity, food habits, smoking history, etc.) of the participants were recorded. Participants over 18 years old were selected as the target population. After the physical examination, 30,042 participants were selected and their anthropometric indices and laboratory tests were assessed in phases 2 and 3. Written consent forms were completed and signed by all participants during the study’s first phase. Details of the study (setting, questionnaires, sampling method, data collection, and non-response error) have been published previously (33). This study was approved by the Ethics Committee of Shiraz University Medical Sciences (IR.SUMS.SCHEANUT.REC.1400.035).

### Measurements

Weight and height were assessed using the standard scale and ruler (33). Body mass index (BMI) was calculated as weight (35) divided by height (square in meter), then classified into normal weight (BMI: 18.5–24.9 kg/m^2^), underweight (BMI < 18.5 kg/m^2^), overweight (BMI: 25–29.9 kg/m^2^), and obese (BMI ≥ 30 kg/m^2^) (36).

We calculated the wealth index (WI) using the principal component analysis (PCA) method. Having car, home, bathroom, kitchen, oven, central heating system, freezer, refrigerator, phone, personal computer, TV, mobile phone, internet, washing machine, vacuum cleaner, dishwasher, and air cooler, also access to water and gas pipelines were included in PCA. Kaiser–Mayer–Olkin (KMO) and Bartlett’s tests were used for data reduction. Then, WI was categorized into quintiles (the first quintile presented the poorest category of income rank, and the last quintile showed the wealthiest one) (36).

To assess the participants’ physical activity level, a validated Global Physical Activity Questionnaire (GPAQ), which contains 16 questions based on the frequency, duration, and intensity of the respondent’s physical activity was used (33, 37). Then, the collected data was altered to Metabolic Equivalent of Tasks (METs)-min per week (33).

### Dietary factors

Intake of fruits were extracted from the following question: “How many servings of fruits do you usually eat each day?” Also, similar questions were asked for vegetables or dairy products intakes. A cup of diced or one medium-size fruit, a cup of raw or half a cup of cooked vegetables, and one cup of dairy products were defined as one serving (33). Then, fruit, vegetable, and dairy intake was categorized as follows:

(1) Fruits: less than 1 servings, 1 servings, 2 servings, and more than 2 servings per day
(2) Vegetables: less than 2 servings, 2 servings, 3 servings, and more than 3 servings per day
(3) Dairy products: less than 1 servings, 1 servings, 2 servings, and more than 2 servings per day.

### Statistical analysis

Baseline characteristics of the study participants across the BMI category were shown as percentage, mean ± standard deviation or median [interquartile range (IQR)]. We used multinomial logistic regression to evaluate the odds ratios (OR) and 95% confidence intervals (CI) to association between the exposures (consumption of dairy products, fruits, and vegetables) and outcome (BMI category) in the crude and adjusted models. We included gender, age, education, physical activity, area of residency, medication, marital status, occupation, smoking history, and sociodemographic variables as confounder in the adjusted model. SPSS (version 23, SPSS Inc., Chicago IL, USA) was used for data analysis and R software (version 3.0.2) for all figures depiction. *P*-values less than 0.05 was considered as statistically significant.

## Results

**FIGURE 1:**
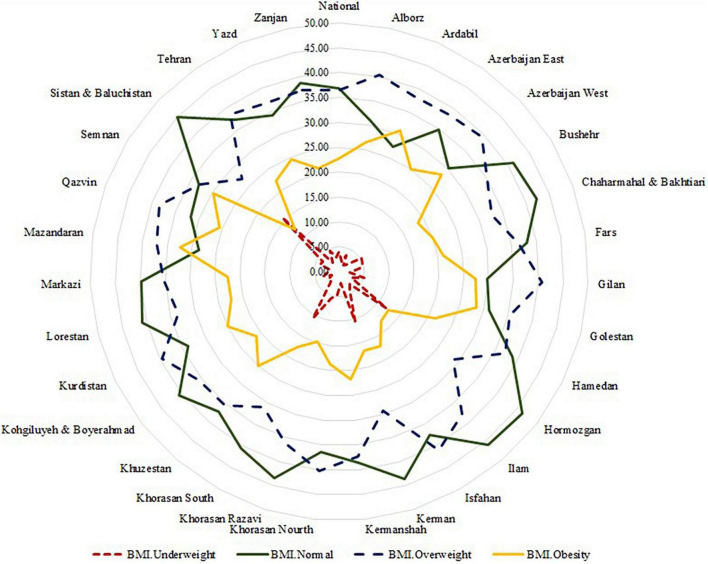
The percent of national and provincial prevalence of underweight, normal, overweight, and obesity from the Stepwise approach to surveillance (STEPS) 2016 study in Iran. The red line (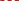) is expressed as a percentage of underweight, green line (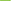) prevalence of normal weight, blue line (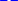) prevalence of overweight and yellow line (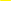) prevalence of obesity across Iran’s provinces. In the provincial level, lowest prevalence of overweight and obesity was seen in Sistan and Baluchistan (26.91% and 12.26, respectively) and highest was seen in Isfahan (40.97%) and Mazandarazn (32.28%), respectively.

According to Table 1, mean age of the participants with normal weight, underweight, overweight, and obese was 41.4, 39.1, 45.9, and 48 years, respectively (*P* < 0.001). Gender, area of residency, medication, smoking and marital status, occupation and wealth index were significantly different between BMI classification (*P* < 0.001 for all). Also, BMI, physical activity and education mean were significantly different between BMI groups (*P* < 0.001 for all). National prevalence of BMI category are shown in Figure 1. At the national level, prevalence of underweight, overweight, and obesity were 4.4, 36.5, and 22.7%, respectively. In the provincial level, lowest and highest prevalence of overweight was seen in Sistan and Baluchistan (26.91%) and Isfahan (40.97%). In the term of obesity, lowest and highest prevalence was observed in Sistan and Baluchistan (12.26%) and Mazandarazn (32.28%), respectively, (national and provincial of average BMI are shown in the Supplementary Figures 1, 2).

**TABLE 1:** Socio-demographic characteristics of the study population by BMI categories.

Variables	Normal weight (10,711)	Underweight (1,176)	Overweight (10,624)	Obesity (6,612)	Total (29,123)	*P*-value
Gender, %^1^						<**0.001**
Male/Female	55.5/44.5	52.3/47.7	50.6/59.4	32.3/67.7	48.3/51.7	
Area of residency, %^1^						<**0.001**
Urban/Rural	66.0/34.0	54.8/45.2	74.1/25.9	74.9/25.1	70.5/29.5	
Medication, % ^1^						<**0.001**
Yes/No	24.5/75.5	24.6/75.4	27.5/72.5	33.1/66.9	27.6/72.4	
Smoking, % ^1^						<**0.001**
Yes/No	23.2/76.8	28.6/31.4	19.7/80.3	15.1/84.9	20.3/79.7	
Marital, % ^1^						<**0.001**
Married/Single	77.2/22.8	65.4/34.6	89.5/10.5	93.9/6.1	85.0/15.0	
Occupation, %^1^						<**0.001**
Employee	8.2	3.6	10.1	6.9	8.4	
Worker	8.2	5.1	6.3	3.5	6.3	
Self-paid	27.5	26.1	25.0	17.7	24.3	
housekeeper	33.2	31.0	41.3	59.8	42.1	
Other	23.0	34.2	17.3	12.0	18.9	
Wealth index, %^1^						<**0.001**
Poorest	21.3	26.6	19.5	16.6	19.8	
Poor	20.1	20.5	20.5	20.0	20.3	
Moderate	19.3	18.8	20.0	21.1	20.0	
Rich	20.4	18.9	19.6	20.5	20.1	
Richest	18.9	15.2	20.3	21.7	19.9	
Age, year^2^	41.4 ± 17.1	39.1 ± 18.9	45.9 ± 15.2	48.0 ± 13.8	44.4 ± 16.1	<**0.001**
BMI, kg/m^2^	22.4 ± 1.7	17.2 ± 1.1	27.3 ± 1.4	33.6 ± 3.3	26.5 ± 5.1	<**0.001**
Physical activity, MET/min/week ^3^	480.0 (2400.0)	480.0 (1960.0)	480.0 (1920.0)	240.0 (1560.0)	420. (1920.0)	<**0.001**
Education, year^3^	10.0 (7.0)	9.0 (8.0)	9.0 (7.0)	6.0 (9.0)	9.0 (7.0)	<**0.001**

BMI, body mass index; MET, metabolic of task. Values are reported mean ± SD or median (IQR) and percentage. ^1^Using chi-square test for categorical variables. ^2^Using one-way ANOVA for normal continuous variables. ^3^Using Kruskal–Wallis for abnormal continuous variables. Normal weight: BMI 18.5–24.9 kg/m^2^, underweight: BMI < 18.5 kg/m^2^, overweight: BMI 25–29.9, and obesity: BMI ≥ 30 kg/m^2^. Bold values represent the significant values.

**FIGURE 2:**
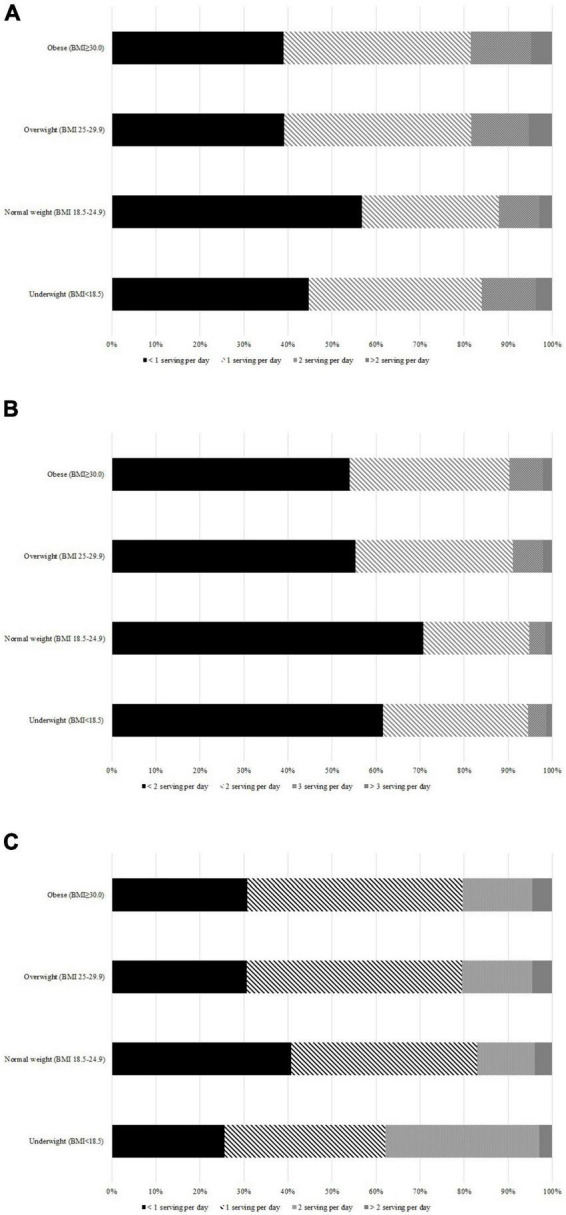
The percent of fruits, vegetables and dairy products serving per day based on the body mass index (BMI) classification from the Stepwise approach to surveillance (STEPS) 2016 study in Iran. **(A)** The percent of fruits consumptions from the STEPS 2016 study in Iran. **(B)** The percent of vegetables consumptions from the STEPS 2016 study in Iran. **(C)** The percent of dairy products consumptions from the STEPS 2016 study in Iran.

Distribution of the intake of fruits, vegetables, and dairy products based on the BMI classification is presented in Figures 2A–C. In the whole population, 41.9% consumed less than 1 and 4.5% more than 2 servings of fruits per day, 57.6% consumed less than 2 and 1.5% more than 3 servings of vegetables per day, and 32.4% consumed less than 1 and 4.2% more than 2 servings of dairy products per day. The subnational information has been presented in Supplementary Figures 3–5.

As shown in Table 2, in the crude model, participants who consumed 1, 2, and more than 2 servings of fruits per day had 37% (OR = 0.63; 95% CI: 0.55, 0.73, *P* < 0.001), 21% (OR = 0.79; 95% CI: 0.69, 0.90, *P* = 0.001), and 22% (OR = 0.78; 95% CI: 0.68, 0.91, *P* = 0.002) lower odds of being overweight compared to less than one serving per day. Also, we observed 44% (OR = 0.56; 95% CI: 0.46, 0.70, *P* < 0.001), 32% (OR = 0.68; 95% CI: 0.55, 0.84, *P* < 0.001), and 31% (OR = 0.69; 95% CI: 0.55, 0.88, *P* = 0.002) lower odds of being overweight among the participants who consumed 2, 3, and more than 3 servings of vegetables per day compared to less than 2 serving per day, respectively. Furthermore, regarding dairy products, the odds of being overweight was 21% lower among people who consumed 1 serving of dairy products per day compared to less than one serving per day (OR = 0.79; 95% CI: 0.69, 0.91, *P* = 0.002). Moreover, in the crude model, participants who consumed 1 serving of fruit per day had 31% (OR = 0.69; 95% CI: 0.59, 0.80, *P* < 0.001) lower odds of obesity in comparison to less than one serving per day, respectively. Also, compared to the participants who consumed less than 2 servings of vegetables per day, 27% (OR = 0.63; 95% CI: 0.49, 0.80, *P* < 0.001) lower odds of obesity was observed among participants who consumed 2 servings of vegetables per day. For the dairy products consumptions, participants who consumed 1, 2, and more than 2 serving per day compared to less than one serving per day had 25% (OR = 0.75; 95% CI: 0.64, 0.88, *P* < 0.001), 17% (OR = 0.83; 95% CI: 0.71, 0.97, *P* = 0.021), and 17% (OR = 0.83; 95% CI: 0.70, 0.98, *P* = 0.031) lower odds of obesity, respectively.

**TABLE 2:** Association between intake of fruits, vegetables and dairy products with body mass index (BMI) in the crude model.

Fruits serving per day							
**Variables**	**<1 serving**	**1 serving**	***P*-value**	**2 serving**	***P*-value**	**>2 serving**	***P*-value**
Underweight vs. normal weight*	Ref.	**1.60 (1.12, 2.28)**	**0**.**009**	0.99 (0.69, 1.42)	0.970	0.93 (0.62, 1.39)	0.738
Overweight vs. normal weight	Ref.	**0.63 (0.55, 0.73)**	<**0**.**001**	**0.79 (0.69, 0.90)**	**0.001**	**0.78 (0.68, 0.91)**	**0**.**002**
Obesity vs. normal weight	Ref.	**0.69 (0.59, 0.80)**	<**0**.**001**	0.85 (0.73, 1.00)	0.053	0.88 (0.74, 1.05)	0.172
**Vegetables serving per day**							
**Variables**	**<2 serving**	**2 serving**	***P*-value**	**3 serving**	***P*-value**	**>3 serving**	***P*-value**
Underweight vs. normal weight	Ref.	1.05 (0.64, 1.73)	0.827	0.67 (0.40, 1.10)	0.120	0.57 (0.32, 1.02)	0.061
Overweight vs. normal weight	Ref.	**0.56 (0.46, 0.70)**	<**0**.**001**	**0.68 (0.55, 0.84)**	<**0.001**	**0.69 (0.55, 0.88)**	**0**.**002**
Obesity vs. normal weight	Ref.	**0.63 (0.49, 0.80)**	<**0**.**001**	0.79 (0.62, 1.01)	0.067	0.90 (0.69, 1.18)	0.479
**Dairy products serving per day**							
**Variables**	**<1 serving**	**1 serving**	***P*-value**	**2 serving**	***P*-value**	**>2 serving**	***P*-value**
Underweight vs. normal weight	Ref.	1.14 (0.83, 1.56)	0.412	0.83 (0.60, 1.14)	0.253	0.78 (0.55, 1.10)	0.167
Overweight vs. normal weight	Ref.	**0.79 (0.69, 0.91)**	**0**.**002**	0.88 (0.77, 1.01)	0.087	0.90 (0.77, 1.04)	0.174
Obesity vs. normal weight	Ref.	**0.75 (0.64, 0.88)**	<**0**.**001**	**0.83 (0.71, 0.97)**	**0.021**	**0.83 (0.70, 0.98)**	**0**.**031**

*Reference category. Normal weight: BMI 18.5–24.9 kg/m^2^, underweight: BMI < 18.5 kg/m^2^, overweight: BMI 25–29.9 and obesity: BMI ≥ 30 kg/m^2^. These values are odds ratio (95% CIs). Obtained from multinomial regression. Bold values represent the significant values.

After adjusting for potential confounders, we observed 41% (OR = 0.59; 95% CI: 0.50, 0.68, *P* < 0.001), 27% (OR = 0.73; 95% CI: 0.62, 0.84, *P* < 0.001), and 26% (OR = 0.74; 95% CI: 0.63, 0.87, *P* < 0.001) lower odds of being overweight, and 46% (OR = 0.54; 95% CI: 0.46, 0.64, *P* < 0.001), 29% (OR = 0.71; 95% CI: 0.60, 0.84, *P* < 0.001), and 21% (OR = 0.79; 95% CI: 0.65, 0.95, *P* = 0.014) decrease in obesity odds among the participants who consumed 1, 2, and more than 2 servings of fruits per day in comparison to less than one serving per day, respectively. Also, we observed participants who consumed 1, 2, and more than 2 servings of dairy products per day in comparison to less than one serving per day had 31% (OR = 0.69; 95% CI: 0.58, 0.81, *P* < 0.001), 23% (OR = 0.77; 95% CI: 0.65, 0.91, *P* = 0.002), and 21% (OR = 0.79; 95% CI: 0.67, 0.94, *P* = 0.011) lower odds of being overweight and 47% (OR = 0.53; 95% CI: 0.44, 0.64, *P* < 0.001), 36% (OR = 0.64; 95% CI: 0.53, 0.77, *P* < 0.001), and 32% (OR = 0.68; 95% CI: 0.56, 0.83, *P* < 0.001) lower odds of obesity, respectively. In addition, compared to participants who consumed less than 2 servings of vegetables per day, participants who consumed 2, 3, and more than 3 servings had 40% (OR = 0.60; 95% CI: 0.47, 0.76, *P* < 0.001), 29% (OR = 0.71; 95% CI: 0.56, 0.90, *P* = 0.006), and 26% (OR = 0.74; 95% CI: 0.57, 0.96, *P* = 0.027) lower odds of being overweight, respectively. Furthermore, we observed 36% lower odds of obesity among participants who ate 2 servings of vegetables per day compared to less than 2 serving per day (OR = 0.64; 95% CI: 0.49, 0.84, *P* = 0.002) (Table 3).

**TABLE 3:** Association between intake of fruits, vegetables and dairy products with body mass index (BMI) in the adjusted model.

Fruits serving per day							
**Variables**	**<1 serving**	**1 serving**	***P*-value**	**2 serving**	***P*-value**	**>2 serving**	***P*-value**
Underweight vs. normal weight*	Ref.	1.46 (0.98, 2.16)	0.061	1.02 (0.68, 1.52)	0.886	0.95 (0.61, 1.48)	0.851
Overweight vs. normal weight	Ref.	**0.59 (0.50, 0.68)**	<**0**.**001**	**0.73 (0.62, 0.84)**	<**0**.**001**	**0.74 (0.63, 0.87)**	<**0**.**001**
Obesity vs. normal weight	Ref.	**0.54 (0.46, 0.64)**	<**0**.**001**	**0.71 (0.60, 0.84)**	<**0**.**001**	**0.79 (0.65, 0.95)**	**0**.**014**
**Vegetables serving per day**							
**Variables**	**<2 serving**	**2 serving**	***P*-value**	**3 serving**	***P*-value**	**>3 serving**	***P*-value**
Underweight vs. normal weight	Ref.	0.97 (0.56, 1.67)	0.978	0.67 (0.38, 1.17)	0.176	0.60 (0.32, 1.14)	0.128
Overweight vs. normal weight	Ref.	**0.60 (0.47, 0.76)**	<**0**.**001**	**0.71 (0.56, 0.90)**	**0**.**006**	**0.74 (0.57, 0.96)**	**0**.**027**
Obesity vs. normal weight	Ref.	**0.64 (0.49, 0.84)**	**0**.**002**	0.82 (0.62, 1.08)	0.174	0.98 (0.73, 1.32)	0.906
**Dairy products serving per day**							
**Variables**	**<1 serving**	**1 serving**	***P*-value**	**2 serving**	***P*-value**	**>2 serving**	***P*-value**
Underweight vs. normal weight	Ref.	1.08 (0.75, 1.55)	0.659	0.83 (0.57, 1.19)	0.325	0.74 (0.50, 1.10)	0.143
Overweight vs. normal weight	Ref.	**0.69 (0.58, 0.81)**	<**0**.**001**	**0.77 (0.65, 0.91)**	**0**.**002**	**0.79 (0.67, 0.94)**	**0**.**011**
Obesity vs. normal weight	Ref.	**0.53 (0.44, 0.64)**	<**0**.**001**	**0.64 (0.53, 0.77)**	<**0**.**001**	**0.68 (0.56, 0.83)**	<**0**.**001**

*Reference category. Normal weight: BMI 18.5–24.9 kg/m^2^, underweight: BMI < 18.5 kg/m^2^, overweight: BMI 25–29.9 and obesity: BMI ≥ 30 kg/m^2^. Adjusted model: for gender, age, education, physical activity, area of residency, medication, marital status, occupation, smoking history and wealth index. These values are odds ratio (95% CIs). Obtained from multinomial regression. Bold values represent the significant values.

## Discussion

The current cross-sectional study on 29,123 Iranian adults showed that the odds of being overweight and obese decreased by 26% and 21%, respectively, with the consumption of more than 2 servings of fruits per day. Also, consuming 2 servings of vegetables per day was associated with 40% reduction in overweight and 36% obesity. In addition, consuming more than 2 servings of dairy products per day caused 21% reduction in overweight and 32% in obesity. These findings indicated that more consumption of fruits, vegetables, and dairy products could be associated with lower odds of overweight and obesity.

A systematic review study of the longitudinal and experimental studies among adults showed an inverse relationship between FVs consumption and obesity (7). In these investigations, mainly in experimental studies, it is unclear whether this association is due to high consumption of FVs or multiple changes in lifestyle-related behaviors including consumption of low-energy foods or being more physically active (7). Also, studies reported that the consumption of dairy products may exert beneficial effects on body weight in both children and adults (38, 39).

A cross-sectional study on adults in the United States showed an inverse relationship between the intake of FVs and BMI (40). Also, a study by Wall et al. (41) indicated that adolescents who consumed more FVs had lower BMI. Moreover, a cohort study revealed a negative association between FVs consumption and BMI among Chinese men (15). Further, a systematic review by Schlesinger et al. (42) showed a negative association between FVs and the risk of overweight and obesity. In contrast, in a large-scale study conducted in Australia, more intake of FVs was associated with overweight and obesity among women (43), which could be attributed to the over-consumption of nutritious foods as well as high-energy and low-nutrient foods. However, another study found that FVs intake may manipulate the weight status differently, depending on the individual characteristics (44).

Beneficial effects of FVs consumption on weight control could be justified by reduced consumption of energy-dense foods (7). A study on adults showed that those who consumed more fruits received less energy over time. This means that increasing FVs intake without intentional restriction of energy intake or high-calorie foods may be replaced for high-calorie foods (45). Furthermore, weight control could be affected by consumption of fiber-rich foods such as fruits and vegetables through different mechanisms, including decreased energy intake, excretion of bile acids and possibly mobilization of fat stores to increase their hepatic synthesis, also increased gastrointestinal motility which increases postprandial energy expenditure (46). In general, FVs consumption may affect body weight due to various reasons such as having low glycemic index, low-fat content, and low energy density, also reducing the eating speed (47).

The present study’s findings demonstrated an inverse relationship between dairy product consumption and BMI, which is in line with some previous studies. A study conducted on over 16 years old adults in Tehran Lipid and Glucose Study (TLGS) showed an inverse relationship between dairy intake and BMI (48). Also, a study on French adults demonstrated that dairy consumption, especially yogurt and milk, was inversely related to body weight (49). Moreover, a study by Trichia et al. (50) found that consumption of more fermented and low-fat dairy products was associated with lower BMI and body weight. In contrast, Gunther et al. (51) reported that increasing consumption of dairy products did not affect body weight among healthy women. Also, a meta-analysis showed no effect of dairy consumption on weight loss without energy restriction or in long-term studies. However, their results suggested that dairy products may exert little benefit in facilitating weight loss during calorie restriction (24). There are various mechanisms through which dairy products may influence body weight, including increasing calcium intake, which stimulates lipolysis and reduces lipogenesis (52). Calcium could bind to fatty acids in the intestine and prevent their absorption by forming soap (53), so, reduces the calorie intake. However, high doses of calcium (1,800 mg of calcium per day) are required for the relatively small energy loss through the stool. Therefore, this mechanism cannot fully explain the effects of calcium on weight control (49). Besides, conjugated linoleic acid content of dairy products may play a role in regulating lipid metabolism and lipogenesis (54).

One of the strengths of this study was the large number of people participating in the research, which increased the statistical power of the findings. Another strength of the study was the control of confounding factors, such as physical activity, sex, age, and wellbeing factors. The current study also had some limitations. First of all, due to the cross-sectional nature of the study, it was not possible to speak out the effects of FVs and dairy products on body weight. We also could not separate high-fat dairy products from low-fat ones due to the unavailability of the data. In addition, as the energy intake was not reported in STEPS study, we were not able to adjust our results for total energy intake, which is an important variable when examining body weight changes.

## Conclusion

The present study supports a potentially beneficial role of FVs and dairy product consumption on BMI. Indeed, consumption of FVs and dairy products have been shown to influence the maintenance of a healthy weight status. Since obesity is increasing worldwide, including in Iran, promoting healthy eating habits such as FVs and dairy product consumption is recommended to prevent obesity. Further studies are needed to confirm the findings of our study.

## Data availability statement

The data that support the findings of this study are available on request from the corresponding author.

## Ethics statement

The studies involving human participants were reviewed and approved by the Ethic Committee of Shiraz University Medical Sciences Center (IR.SUMS.SCHEANUT. REC.1400.035). The patients/participants provided their written informed consent to participate in this study.

## Author contributions

MN and ZS designed the study, involved in the analysis, and draft the manuscript. SF was involved in the design of the study, analysis of the data, and critically reviewed the manuscript. All authors read and approved the final manuscript.

## Supplementary material

Supplementary Figure 1 The distribution of average BMI in the provincial level from the STEPS 2016 study in Iran.

Supplementary Figure 2 The distribution of average BMI in the county level from the STEPS 2016 study in Iran.

Supplementary Figure 3 The percent of provincial distribution of more than 2 servings of fruit consumption from the STEPS 2016 study in Iran.

Supplementary Figure 4 The percent of provincial distribution of more than 3 servings of vegetable consumption from the STEPS 2016 study in Iran.

Supplementary Figure 5 The percent of provincial distribution of more than 2 servings of dairy products consumption from the STEPS 2016 study in Iran.
