# Supplementary material for: The relationship between intake of fruits, vegetables and dairy products with overweight and obesity in a large sample in Iran: Findings of STEPS 2016
Source: Front Nutr. 2023 Jan 17;9:1082976. doi: 10.3389/fnut.2022.1082976 (PMC9886861; doi:10.3389/fnut.2022.1082976)
Supplement: Supplementary Figure 1 — The distribution of average BMI in the provincial level from the STEPS 2016 study in Iran. [file Table_1.docx]

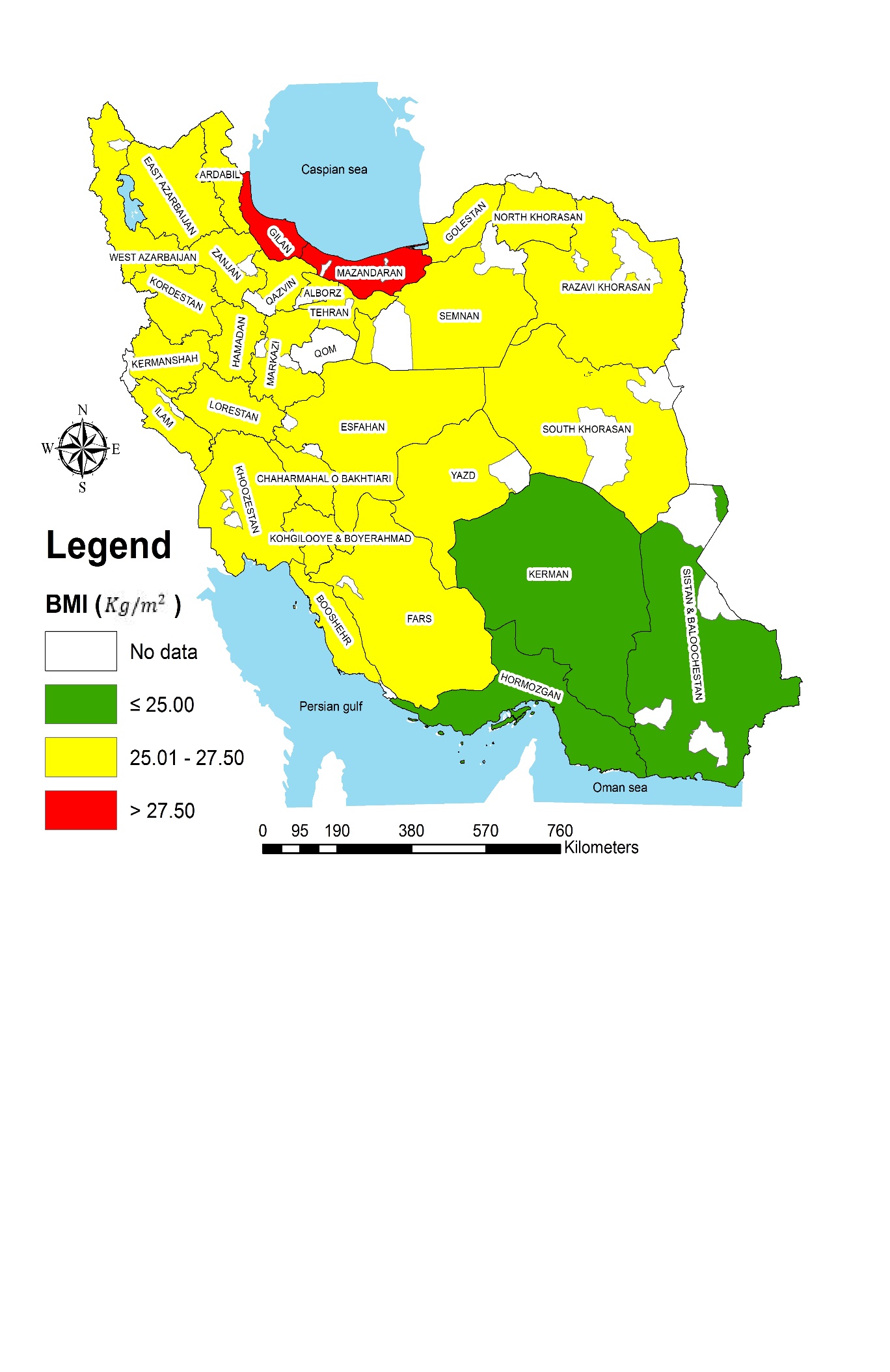


**Figure A.** The distribution of average BMI in the provincial level from the STEPS 2016 study in Iran.


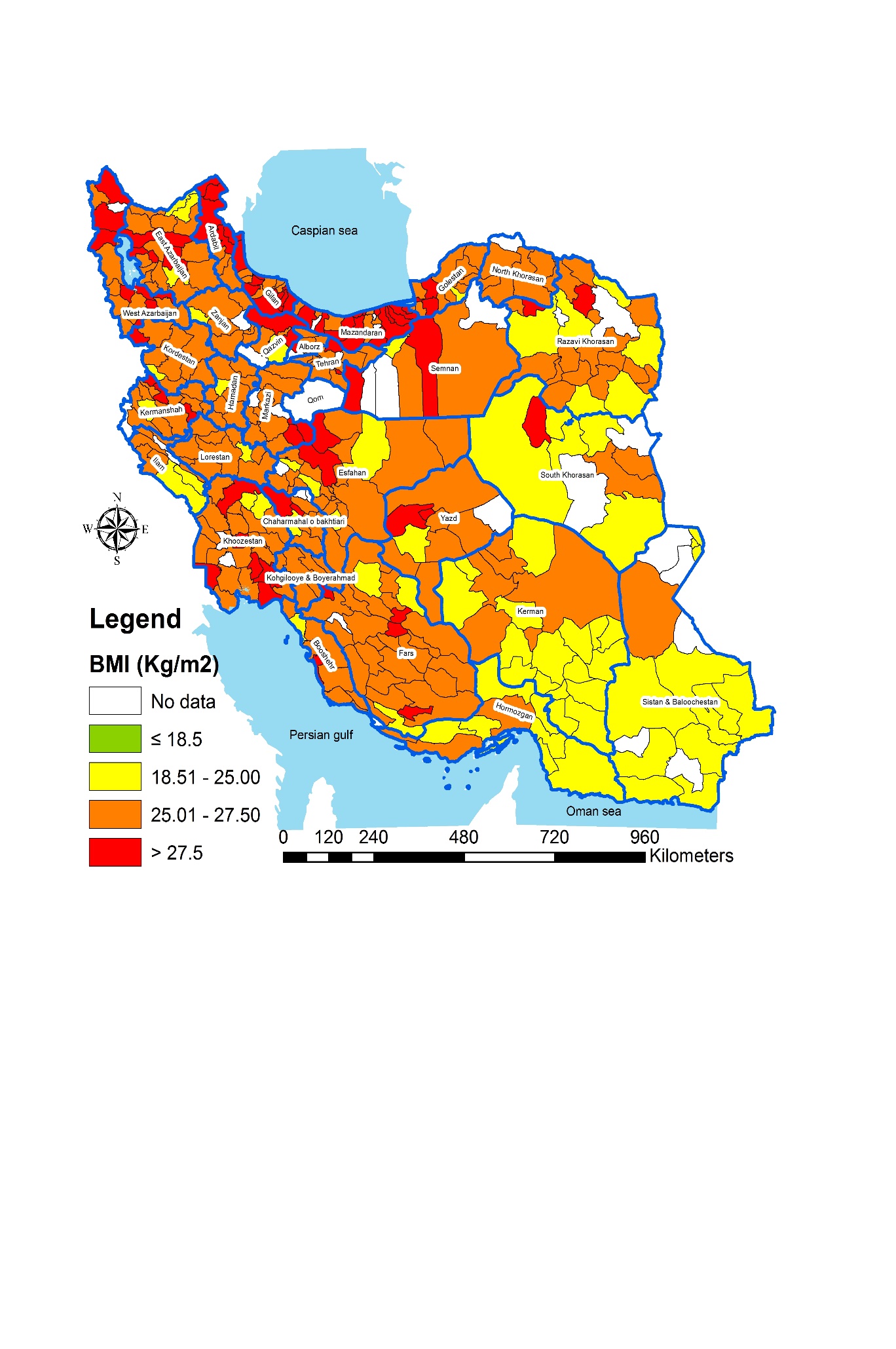


**Figure B.** The distribution of average BMI in the county level from the STEPS 2016 study in Iran.


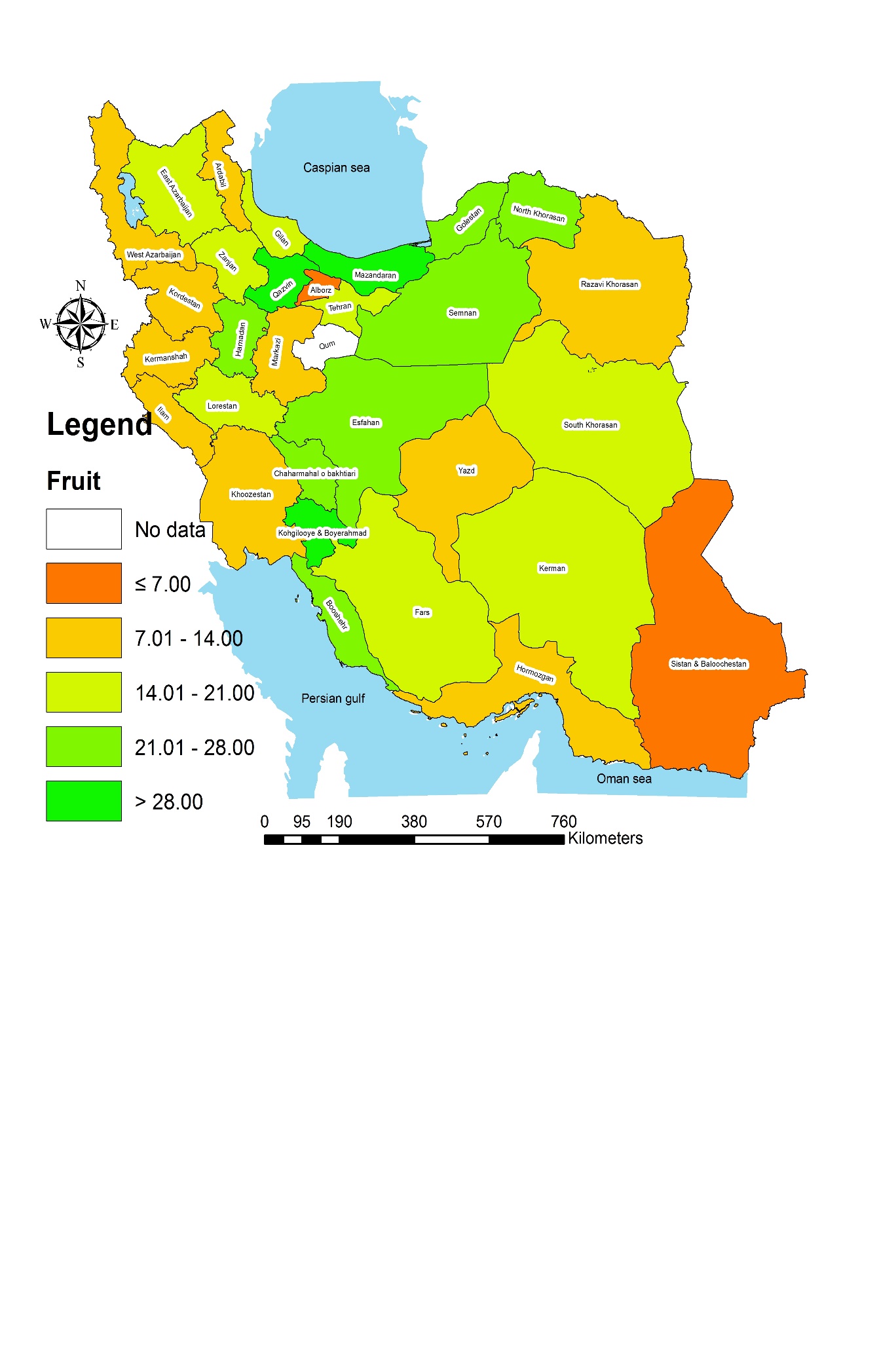


**Figure C.** The percent of provincial distribution of more than 2 servings of fruit consumption from the STEPS 2016 study in Iran.


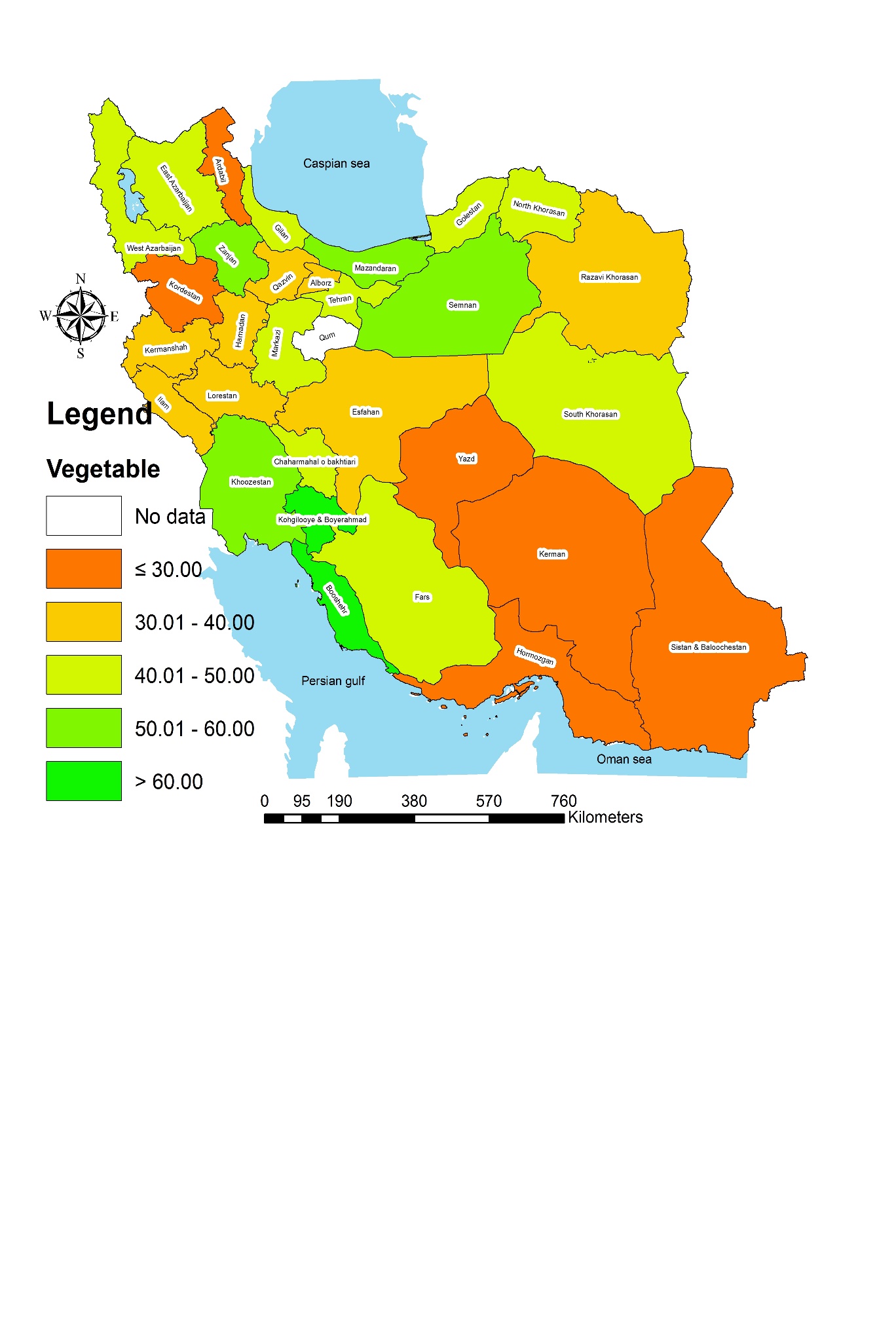


**Figure D.** The percent of provincial distribution of more than 3 servings of vegetable consumption from the STEPS 2016 study in Iran.


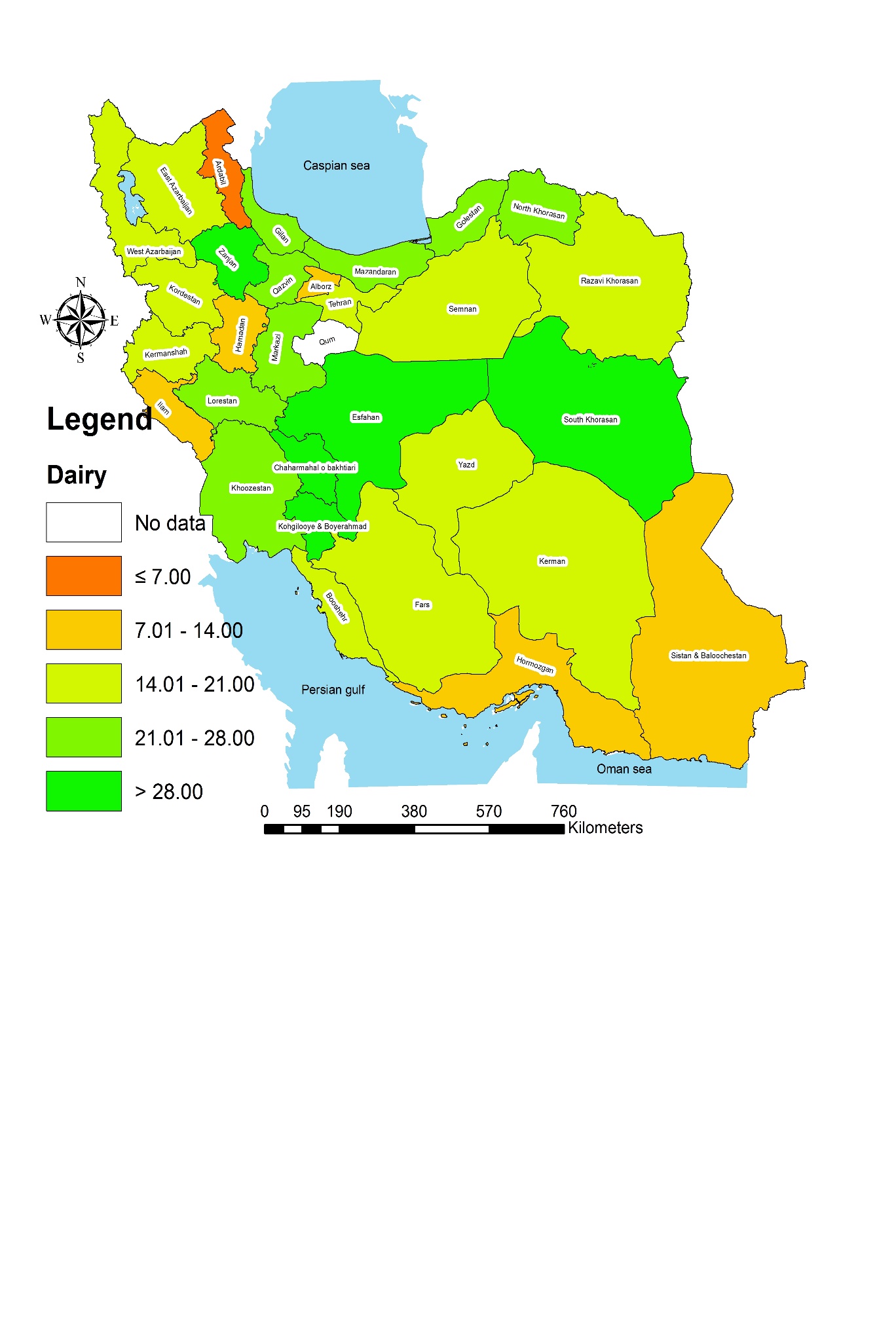


**Figure E.** The percent of provincial distribution of more than 2 servings of dairy products consumption from the STEPS 2016 study in Iran.
